# Advanced Plastic Waste Recycling—The Effect of Clay on the Morphological and Thermal Behavior of Recycled PET/PLA Sustainable Blends

**DOI:** 10.3390/polym15143145

**Published:** 2023-07-24

**Authors:** Maria-Paraskevi Belioka, Georgia Markozanne, Kiriaki Chrissopoulou, Dimitrios S. Achilias

**Affiliations:** 1 Lab of Polymer and Color Chemistry and Technology, Department of Chemistry, Aristotle University of Thessaloniki, 54124 Thessaloniki, Greece; mpelioka@chem.auth.gr; 2 Institute of Electronic Structure and Laser, Foundation for Research and Technology—Hellas, 70013 Heraklion, Crete, Greece; markozanne@iesl.forth.gr (G.M.); kiki@iesl.forth.gr (K.C.)

**Keywords:** PET/PLA blends, nanocomposite polymers, nanoclay, plastic waste, recycling, circular economy, sustainable plastics, biobased polymers, morphology, thermal degradation

## Abstract

Bio-based polymers such as poly(lactic acid), PLA, are facing increased use in everyday plastic packaging, imposing challenges in the recycling process of its counterpart polyester poly(ethylene terephthalate), PET. This work presents the exploration of the properties of PET/PLA blends with raw materials obtained from recycled plastics. Several blends were prepared, containing 50 to 90% PET. Moreover, multiscale nanocomposite blends were formed via melt mixing using different amounts and types of nanoclay in order to study their effect on the morphology, surface properties, and thermal stability of the blends. The materials were characterized by X-ray diffraction analysis (XRD), thermo-gravimetric analysis (TGA), scanning electron microscopy (SEM), atomic force microscopy (AFM), and differential scanning calorimetry (DSC). The nanoclay was found to exhibit a uniform dispersion in the polymer matrix, presenting mainly intercalated structures with some exfoliated at low loading and some agglomerates at high loading (i.e., 10%). The addition of nanoclay to PET/PLA matrices increased the roughness of the blends and improved their thermal stability. Thermal degradation of the blends occurs in two steps following those of the individual polymers. Contamination of rPET with rPLA results in materials having poor thermal stability relative to rPET, presenting the onset of thermal degradation at nearly 100 °C lower. Therefore, important information was obtained concerning the recyclability of mixed PET and PLA waste. The perspective is to study the properties and find potential applications of sustainable blends of recycled PET and PLA by also examining the effect of different clays in different loadings. Therefore, useful products could be produced from blends of waste polyester.

## 1. Introduction

Every element of life uses plastics, including clothing, electronics, toys, healthcare supplies, food packaging, and many other products. Currently, the fossil fuel industry provides the majority of the feedstocks used to make plastic. The widely available components of gas and oil are well suited to the chemistry of polymers. Over the past 60 years, these sources have been able to deliver dependable, constant feedstocks for the creation of plastics. Plastics have become more and more common in daily life over time, and new technologies are enhancing their performance, but just as gasoline and diesel availability will decline due to rising petroleum and other fossil-based fuel prices or scarcity, so too will plastics made from fossil resources [1,2]. This growing resource shortage highlights the need for alternate techniques for making plastics. Furthermore, given the size of the business and the availability of resources, it would be preferable to identify ways to produce materials that have less of an impact on the environment. The carbon used to make petroleum-based polymers has been trapped in the soil for millions of years. A net increase in greenhouse gases would occur in the atmosphere if this carbon were to be released through the decomposition of plastics or some other process. Plastics can be disposed of in a number of ways, with varying recycling methods, and have a range of useful lifetimes [2].

Today’s ecologically concerned manufacturers increasingly use biopolymers in addition to or instead of petroleum-based polymers, as well as partially or entirely recycled materials, to create their products. The packaging industry uses most of the biopolymers, just like it does with plastics derived from petroleum. However, because of how they work, they only last a limited time (a few weeks on average); thus, they quickly become waste. Biopolymers made up just around 2% of all plastic produced in 2017, although their volume is growing steadily. Petroleum-based polymer recycling is already well-established, and biodegradable polymers can also be recycled biologically (for example, by commercial composting) [3,4,5].

Due to the widespread usage of durable petroleum polymers, it takes a very long time for the waste produced by these polymers to decompose. These days, there is a serious environmental issue due to the indiscriminate usage of polymers derived from petroleum. Biodegradable polymers from renewable sources, such as collagen, keratin, gluten, milk proteins, soy proteins, polysaccharides like starch, cellulose derivatives, chitosan, alginate, and pectins, have been utilized to lessen this issue [6]. Because they are best used in temporary applications like throwaway packaging, agricultural mulch, horticultural pots, etc., these biodegradable polymers have a short lifespan. Additionally, when they are disposed of in the environment, they naturally degrade. Despite their benefits, many of these polymers have weak mechanical properties, low steam and gas barriers, and poor thermal stability, making them unsuitable for other uses. As a result, the general trend is to combine the mechanical, barrier, and thermal capabilities of polymers derived from petroleum with the biodegradability characteristics of renewable polymers to produce polymeric materials with controllable lifetimes. The designed materials must be durable while in use and must degrade quickly when their useful lives are up [7].

Biopolymers can take on a variety of shapes. They can be partially made from renewable resources and synthesized like traditional plastics, as is the case with bio-based poly(ethylene terephthalate), PET [8,9], or they can be entirely made from renewable resources and fall under the traditional plastics classification numbering system 1–6, like poly(lactic acid) (PLA) [2,8]. Traditional petroleum-based plastics can be replaced by biopolymers, which can be made from a wide range of feedstocks, including agricultural goods like corn or soybeans and non-traditional sources like algae or food waste [9,10,11]. Biopolymers can take the place of petroleum-based polymers in almost every application, including packaging, single-use items, and durable goods. According to the ASTM D6400-04 Standard Specification for Compostable Plastics [12], biopolymers are being created with properties including biodegradability and compostability. Biopolymers provide the chance to lessen the demand on fossil fuels for the 17 million tons of plastic that are used each year for packaging and non-durable items, as well as to divert the 14 million tons of plastic trash that would otherwise end up in landfill [2]. Biopolymers are produced from renewable resources, but this does not guarantee that they will perform better than petroleum-based polymers [13]. For this reason, sustainability evaluations like LCAs are carried out to compare and lessen the environmental consequences of biopolymers [2].

PET, a semicrystalline, thermoplastic polyester with high strength and transparency features as well as outstanding barrier properties, is the best packaging material for disposable soft drink bottles. Unfortunately, the majority of these beverage bottles are only used once before being thrown away, which consequently causes significant environmental issues. Recycling PET that has been wasted and obtaining biodegradable PET-based blends are thus effective ways to cut down on resource usage while also protecting the environment. Because packaging polymers might become contaminated during initial use or storage, recycling post-consumer packaging materials for direct food contact packaging applications is not feasible. Things have improved a lot for PET, though, as bottle-to-bottle recycling for post-consumer PET bottles has been established thanks to its inert nature [7,14]. Moreover, PET is a non-biodegradable polymer since it is an aromatic polyester. Numerous efforts have been made thus far to improve its degradability. Blending PET with other linear polyesters, such as PLA, is one strategy employed [7,15,16].

Applications for PLA include films and fibers for various uses, as well as clear and opaque hard plastics for packaging, throwaway items, long-lasting items, and bottles [17,18]. Any starch-rich feedstock might be used to make PLA because it is formed from lactic acid, which is created during the fermentation of dextrose, which is commonly obtained from corn. There are numerous ways to polymerize lactic acid to produce granules that are utilized to generate industrial items. To increase PLA’s heat resistance or durability, it can be combined with synthetic or natural fibers derived from petroleum. The biodegradable and compostable properties of PLA-based plastics can provide a larger range of disposal choices [1,2,19].

Torres-Huerta and colleagues looked at how the biodegradable substances chitosan and PLA affected the heat decomposition of PET. They demonstrated that PLA and PET interacted with PET more strongly than chitosan did. The mix of 10 wt% PLA and 5 wt% chitosan performed the best in terms of degradability [7]. Acar et al., used a non-isothermal thermogravimetry (TGA) technique to investigate the thermal degradation behavior of a PET/PLA blend [20]. The heat breakdown rate of the PET sample containing 50% PLA was shown to be lower than the blend including 10% PLA using the Kissinger kinetic model. Many multifunctional nanomaterials have been employed in recent years to enhance the thermal properties of PET while also enhancing other PET features [20]. Exfoliated graphite (xGnP) was utilized by Bandla and Hanan to increase the thermal stability of PET [21]. They discovered that the PET matrix’s evenly distributed graphite enhanced the material’s thermal and thermo-oxidative stability. The impact of melt mixing parameters on a PET/clay nanocomposite was studied by Davis et al. [22]. Additionally, some reports discuss the application of nanomaterials to enhance the thermal characteristics of PLA and PET/PLA blends. For instance, using a melt mixing technique, Meng and colleagues created PLA/clay nanocomposites. They demonstrated that the PLA matrix’s thermal properties were enhanced by organically modified clay (Cloisite 30B), which had excellent dispersion [23].

Although there are several studies on the morphological and thermal properties of PET and PLA as individual materials, the investigation of their blends is much more limited. Specifically, besides Acar et al. [19], Gere and Czigany [3], and Torres-Huerta et al. [7] discussed above, very few reports have been published dealing with the properties of PET/PLA blends despite their importance in the PET recycling industry. McLauchlin and Ghita [23] explored the effect of PLA on the mechanical properties and crystallization behavior of blends of PET containing 0.5–20% PLA produced by injection molding. TGA confirmed the independent behavior of the two polymers under thermal degradation conditions. Xia et al. [24] studied the thermal, crystalline, and mechanical properties of PET/PLA blends and found that the introduction of small amounts of PLA promoted the crystallization of PET during the injection molding process. The starting decomposition temperature lowered from 412 °C of pure PET to 330 °C at 50% PLA content. Torres-Huerta et al. [25] studied the morphological and mechanical properties of PLA/PET blends processed by melt extrusion. Jafari et al. [26] and Topkanlo et al. [27] studied the crystallization kinetics of PET/PLA blends. The production of PET/PLA polymer blends was the method used in the aforementioned articles to assess how much PLA affected the materials’ morphological and mechanical qualities. The acquired samples were then analyzed using a variety of techniques to determine their morphological and mechanical characteristics, structural compatibility, miscibility, and the relationships among them. Finally, two studies have been published on the miscibility and compatibilization of PET/PLA blends [28,29].

Our goal in this work is to create degradable PET-based nanocomposites with the aid of PLA and the addition of two nanoclays, specifically a commercially available organically modified clay (Cloisite 25A) and a sodium montmorillonite (Na+ MMT). It was thus interesting to compare the impact of these two different types of clay on the properties of the produced nanocomposites, given that both of them have been applied as antioxidant nanocarriers for packaging in the food industry [24]. In order to highlight the quality of these materials and their combination after reuse and recycling, the originality in this work is the utilization of recycled PET and recycled PLA from waste materials. By examining how much the qualities of the composites were enhanced by the addition of various reinforcing agents in various percentages, the study is made more thorough and in-depth. The work is more particularly concerned with the thermal and morphological characterization of these produced systems. The properties of these composite recycled materials were evaluated for this purpose using TGA, XRD, SEM, and AFM techniques. To the best of our knowledge, very few studies have examined the effects of PET/PLA mixes with various clays on physical–chemical, structural, and morphological aspects as well as the rate at which they thermally degrade. The problem has been examined from many angles in this work, and the findings are described in terms of the amounts of recycled biodegradable polymer (PLA) and various clays that were put in the recycled PET matrix during the extrusion process.

## 2. Materials and Methods

### 2.1. Materials of the Blends and the Nanocomposites

The raw materials used were recycled PET (R-PET) and recycled PLA (R-PLA) obtained from discarded plastic bottles and glasses, respectively, after they were washed, dried, and cut into flakes. For preparation of the nanocomposites, a commercial organically modified clay (Cloisite 25A kindly provided by Southern Clay products) was used. This is a montmorillonite modified with quaternary ammonium salt, i.e., dimethyl 2-ethyl hexyl hydrogenated tallow. The cationic exchange capacity (CEC) of the ammonium salt was 95 meq/100 g clay and its d_001_spacing was 1.91 nm [30,31]. The selection of the specific nanoclay was because it was found to have good performance in the formation of nanocomposite materials with various polymers [30,31]. In addition, a sodium-containing natural montmorillonite, Na+MMT, was used, with it having a CEC 92 meq/100 g clay and d_001_ = 1.18 nm.

### 2.2. Preparation of the Blends and the Nanocomposites

Different amounts of R-PLA (i.e.,10, 30, and 50 wt%), together with commercial PET or R-PET were hand mixed prior to the extrusion process to achieve the following weight ratios of PET to PLA: 90/10, 70/30, and 50/50.Blends with a filament shape (~1 mm indiam. × 200 cm length) were obtained. Furthermore, for preparation of the nanocomposites, Cloisite 25A was added in amounts relative to the blend equal to 1, 5, and 10 wt% and sodium montmorillonite at only 5% wt%. More specifically, the 15 different blends were compounded in the melt state with a co-rotating twin-screw extruder. All extruded blends were immediately cooled at room temperature. The temperature profile of the extruder (from hopper to die) was 260 °C and four heating zones: feeding (225 °C), compression (237.5 °C), distribution (260 °C), and the extrusion die (225 °C). The rotational speed of the extruder screws was 60 rpm. To avoid any hydrolytic degradation, all samples were dried in a vacuum oven for 24 h before use. The compositions and code names of the prepared samples are shown in Table 1.

### 2.3. Morphological and Thermal Measurements

Samples of the blends and nanocomposite blends were examined by scanning electron microscopy (SEM) (JEOL, mod. JSM-6390LV, JEOL USA Inc., Peabody, MA, USA) working at 20 kV and equipped with an OXFRD INCA EDS analyzer (Abingdon, UK). All surfaces were coated with graphite to avoid charging under the electron beam.

The morphology of the films was investigated by X-ray analysis (XRD). The scanning range was varied from 2*θ* = 2 to 20°, and the scanning speed was 1 deg/min, using a MiniFlex II XRD system from Rigaku Co (Tokyo, Japan) with CuKa radiation (λ = 0.154 nm). X-ray analysis of the films was employed to investigate the structure (intercalated or exfoliated) after incorporation of the nanofillers.

Atomic force microscopy (AFM) was used to estimate the roughness data and the topographical features of the samples. A commercial NT-MDT SOLVER-PRO AFM was employed, and all scans were performed in air, under ambient conditions in the dynamic semi-contact (tapping) mode, using a silicon NT-MDT cantilever (NSG01 Series) with nominal values of 5.1 N/m for the spring constant and a 10 nm radius for the tip. Next, 256 × 256 data points were acquired at a 0.6 Hz scan rate. Topographic (height) and phase images (phase) were recorded simultaneously. At least three different 1 μm × 1 μm areas of each sample were scanned, and the average roughness values (Ra and RMS) of the resulting height images were calculated by image processing software. Ra quantifies the deviation of a real surface from an ideal flat plane.

The thermal degradation characteristics of the materials prepared were examined via thermogravimetric analysis (TGA). Approximately 10 mg of the nanocomposite materials were used for TGA tests on a SDT600 TGA/DTA (TA Instruments, New Castle, DE, USA) apparatus. The tests were initiated at room temperature, and the temperature was increased to 600 °C at a heating rate of 10 °C/min. Nitrogen was used as the purge gas during the test. The change in weight as the temperature increased was recorded.

The thermal properties of the materials produced, such as the melting and glass transition temperature as well as crystallization, were measured by differential scanning calorimetry (DSC). The instrument used was a DSC Spectrum One (Perkin Elmer, Waltham, MA, USA). The samples were heated from ambient temperature to 300 °C at 10 °C/min, held at this temperature for 3 min, and cooled to 20 °C at a rate of 10 °C/min. Indium was used for calibration of the instrument. The glass transition temperature was estimated by the half Cp extrapolation method.

## 3. Results

### 3.1. Morphology of the Blends and the Nanocomposites

#### 3.1.1. SEM Measurements

**Figure 1:**
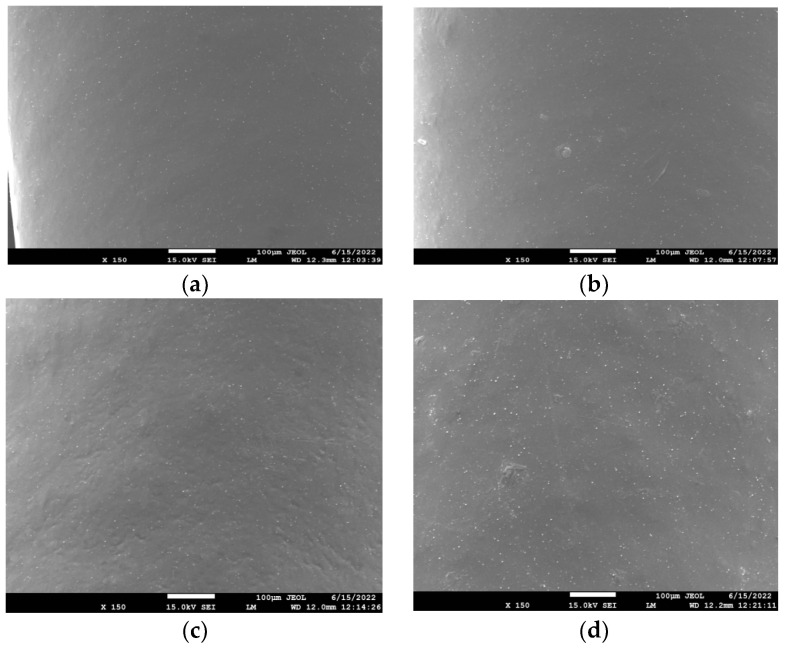
SEM images of the PET/PLA 50/50 blend with (**a**) 1% Cloisite 25A; (**b**) 5 wt% Cloisite 25A; (**c**) 10 wt% Cloisite 25A; and (**d**) 5 wt% Na+MMT.

**Figure 2:**
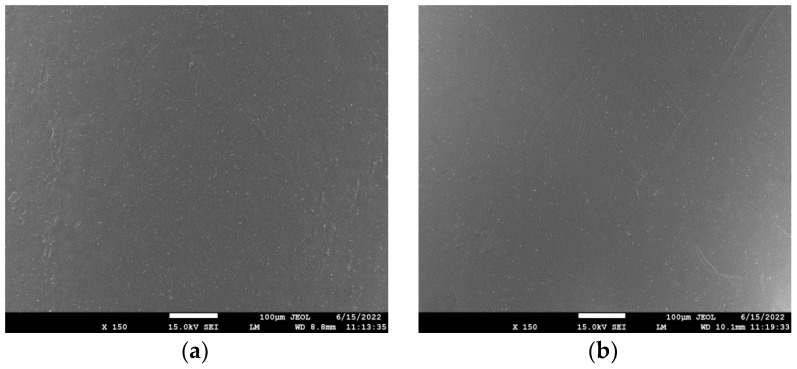

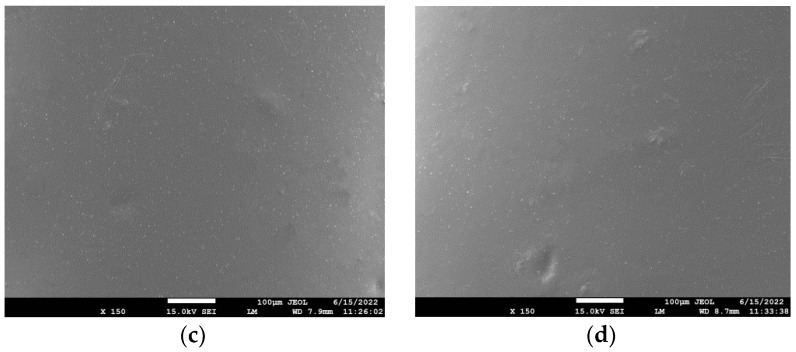
SEM images of the PET/PLA 70/30 blend with (**a**) 1% Cloisite 25A; (**b**) 5 wt% Cloisite 25A; (**c**) 10 wt% Cloisite 25A; and (**d**) 5 wt% Na+MMT.

**Figure 3:**
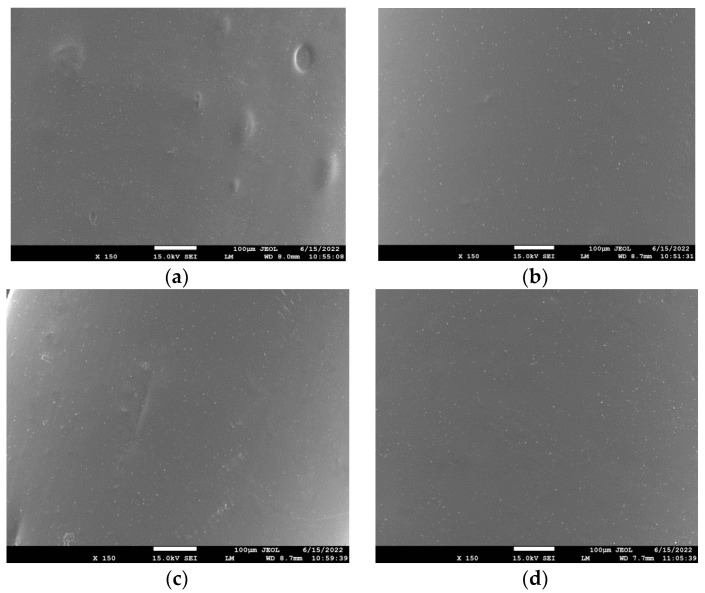
SEM images of the PET/PLA 90/10 blend with (**a**) 1% Cloisite 25A; (**b**) 5 wt% Cloisite 25A; (**c**) 10 wt% Cloisite 25A; and (**d**) 5 wt% Na+MMT.

The dispersion of rPLA in the rPLA/rPET blend as well as that of the nanoclays, Cloisite 25A, and Na+MMT in the composites’ matrix was evaluated using SEM, as shown in Figure 1, Figure 2 and Figure 3 for the blends with 50/50, 70/30, and 90/10 rPLA/rPET, respectively. In the 50-50 blends, it seems that the dispersion of rPLA into rPET is homogeneous. However, in higher amounts of rPET, i.e., in the 90/10 blend (Figure 3), large parts of rPLA seem to be included into the rPET matrix and the blend is not completely homogeneous. Furthermore, with small amounts of nanoclay added (i.e., 1 and 5 wt%), the SEM images show homogeneous samples without any large aggregates of the nanoclay, confirming good dispersion of the nanoclay in the composites. However, some aggregates can be observed in the higher amounts (i.e., 10 wt%) used. In general, the presence of nanoclay aggregates in the polymer matrix is not desirable since it leads to the formation of inhomogeneities in the material. This can lead to inferior mechanical or thermophysical properties (discussed in the next section), which gives an initial indication that such large proportions of clay should be avoided.

#### 3.1.2. Three-Dimensional Images Using AFM

**Figure 4:**
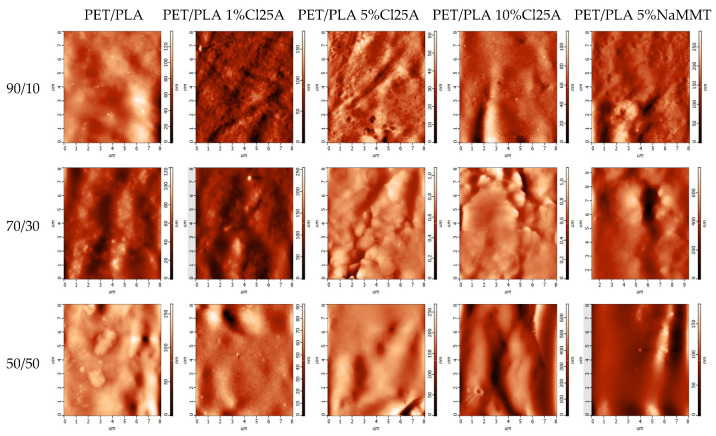
AFM photographs of the PET/PLA blends at various ratios and their nanocomposites with several amounts of Cloisite 25A and Na+MMT.

Furthermore, the topography and roughness of the samples was investigated using atomic force microscopy (AFM). Indicative images of all the nanocomposite blends investigated appear in Figure 4.

Atomic force microscopy is one of the most popular techniques for metrology measurements such as surface roughness due to its ability to quantitatively measure all three dimensions of a surface: lateral and height dimensions at nanoscale resolution; thus, irregularities on the surface can be observed [32]. Unlike other high-resolution microscopic characterization methods, which rely on interactions of electrons with a material, in AFM, there is a mechanical contact between the tip and sample, enabling accurate measurement of sample topography and surface texture. To perform surface roughness measurements, the tapping mode was used. This is a dynamic mode where the tip is oscillated at a resonance frequency, and now the tip gently interacts with the surface at a constant amplitude of oscillation [32]. The two most common roughness parameters that are calculated are the arithmetical mean deviation from the mean and the root mean square (RMS) mean deviation for the mean [33]. For an image where the area is being analyzed, the arithmetical mean is called Sa. Similarly, the RMS roughness is defined as Sq. The values of Sa and Sq estimated for all materials are included in Table 2. It can be observed that increasing the amount of the nanofiller results in the increased roughness of the materials. This increase is more pronounced in the higher amount of 10 wt%. This is expected since the introduction of the nanoclay certainly affects the rather smooth surface of the polymer blend. A similar trend was observed in all different blends. Slightly lower roughness was estimated when the same amount of Na+MMT was used instead of the organomodified one. Furthermore, a slight decrease in roughness was clear with the increasing amount of rPET in the polymer blend but only in the neat blends. No specific trend with the amount of rPET was observed in all nanocomposites.

#### 3.1.3. Crystallinity Measurements via XRD

**Figure 5:**
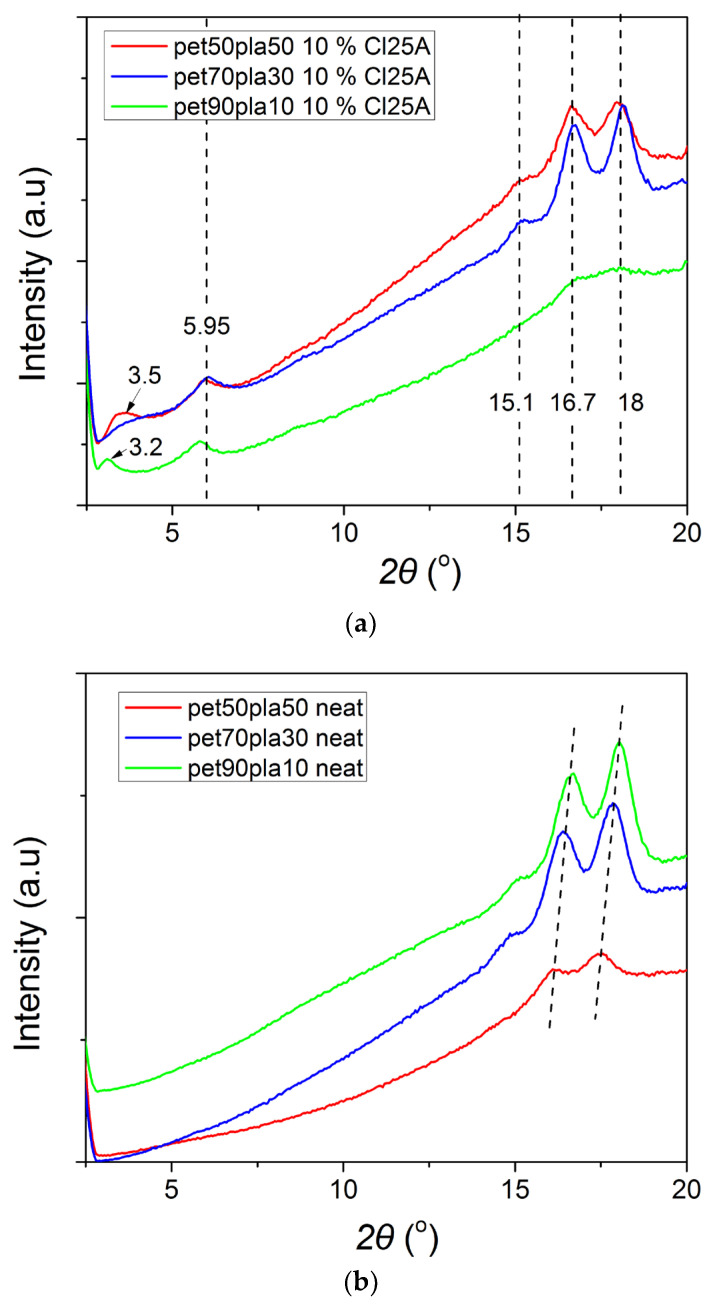
XRD scans of the PET/PLA blends at various ratios (**a**) with 10% Cloisite 25A and (**b**) without the nanoclay.

The crystalline structures of a polymer can be heavily affected by the presence of other compounds in a blend. As a result, it is interesting to find that how the crystalline structure of PET in the blends or the nanocomposites is affected by the presence of the semi-crystalline PLA and/or the nanoclay. To find out the answer, X-ray diffraction (XRD) studies were performed on the neat PET/PLA 90/10, 70/30, and 50/50 blends, as well as on the same blends loaded with 10 wt% of Cloisite 25A, and the resulting XRD patterns are presented in Figure 5. It can be seen that in the neat blends, as the amount of PET increases, a slight shift in the two dominant peaks appears from 16.1 to 16.4 and then 16.6°and from 17.5 to 17.9 and eventually 18.0°. PET is a semi-crystalline polymer with characteristic reflections in the region 1–20°at 16.6° and 17.7°, which are attributed to the (011) and (010) crystallographic planes, respectively [34]. Neat PLA exhibits a very strong diffraction peak at 2*θ* = 16.8° due to diffraction from the (1 1 0) and/or (2 0 0) planes and less intense peaks at 2*θ* = 15.0 and 19.1°, attributed to reflections of the (0 1 0) and (2 0 3) planes, respectively [35]. Therefore, the blends exhibit the two major crystalline reflections at 2*θ* = 16.7 and 18.0° due to diffraction from the (0 1 1) and (0 1 0) planes, although it seems that the crystalline structure is slightly affected as the amount of PET in the blends is increased. The decreasing intensity of the 50/50 neat blend is an indication that the degree of crystallinity of this sample is slightly lower. This is verified in the following section from additional DSC measurements. Moreover, as the amount of PLA is increased, a new distinct peak appears at 15.1°, which is related to the (0 10) crystalline plane of PLA. This is not present in the 90/10 blend. However, what was very interesting was that in the presence of 10% Cloisite 25A, the position of the dominant reflection peaks remains unchanged. Particularly the first peak at 16.7° and the second at 18° together with the small peak at 15.1°are attributed to PLA only without them being affected much by the different amounts of PET in the blend. We hypothesize that the presence of the nanoclay results in a more stable nanocomposite blend presenting a similar crystal structure independent of the amount of PET or PLA. Similar results have been reported in the literature for similar PET/PLA blends [7,26].

**Figure 6:**
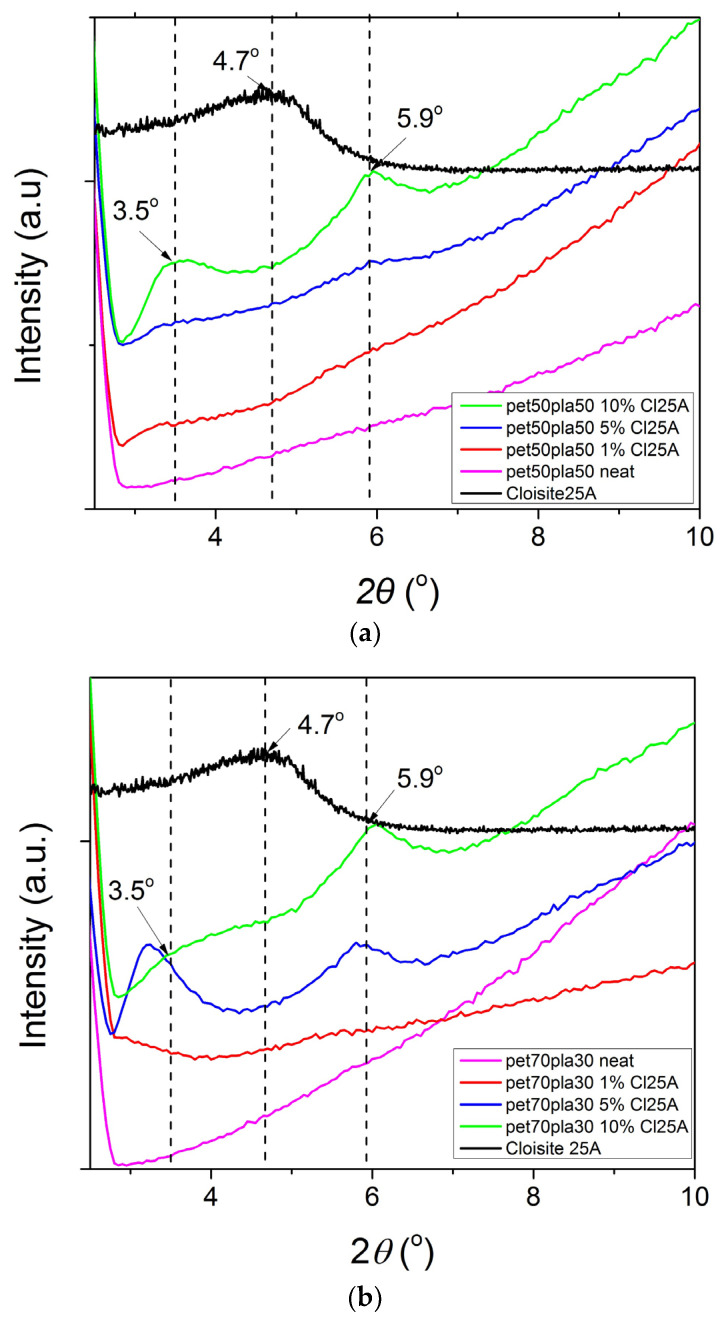

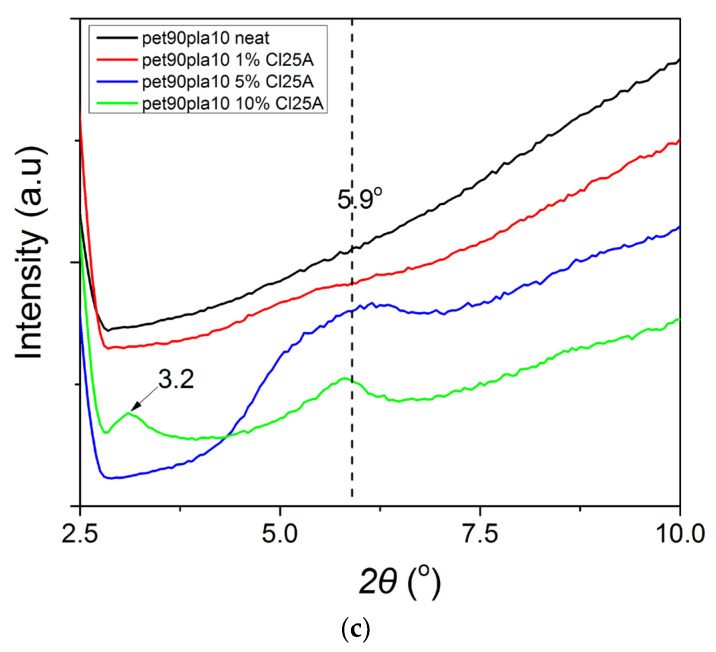
Effect of the amount of the nanoclay Cloisite 25A on theXRD scans of PET/PLA blends with (**a**) 50/50, (**b**) 70/30, and (**c**) 90/10 relative amounts of rPET/rPLA.

Furthermore, it is interesting to study the status of the nanoclay in the polymer matrix. It is known that the platelets of the nanoclay can be either intercalated or exfoliated in the presence of the macromolecular chains. This can be easily identified from XRD measurements at low angles. Therefore, the results are illustrated in Figure 6, focusing only on the range from 2 to 10°. The neat Cloisite 25A diffraction peak was observed at 4.7°, which is equivalent to 1.89 nm, a value similar to that reported by the company that supplied the nanoclay, i.e., 1.86 nm. In most nanocomposite blends, two reflection peaks appear at 3.5° and 5.9°. The first corresponds to a d_001_ spacing of 2.53 nm and the second corresponds to 1.5 nm. The increase in the spacing between the platelets from 1.89 to 2.53 nm is an indication of an intercalated structure. Exfoliated clay would result in the absence of diffraction peaks. However, a decrease in the d-spacing to 1.5 nm is an indication of the presence of clay tactoids (the existence of small-scale agglomerates). The intensity of the peaks of the nanocomposites with 1% clay is lower if any of them reflect on one side with the low amount of filler, but this also indicates that the clay exits in partially intercalated and partially exfoliated structures. In contrast, in the nanocomposite with 10% Cl25A, the peaks are clearly indicating both intercalated structures with clay tactoids. In the 70/30 blend after the addition of 1 or 5% Cloisite 25A, the intercalation seems better since the diffraction peak was observed to be lower at 2*θ* = 3.3°, corresponding to d-spacing of 2.69 nm.

From the above, it can be concluded that the degree of crystallinity of the 50/50 neat blend is slightly lower than that of the 70/30 and 90/10 blends, and that low amounts of nanoclay result in partially exfoliated and intercalated structures, whereas a high amount results, besides to the intercalated structures, to clay tactoids (aggregates) as well.

### 3.2. Thermal Stability of the Blends and the Nanocomposites

**Figure 7:**
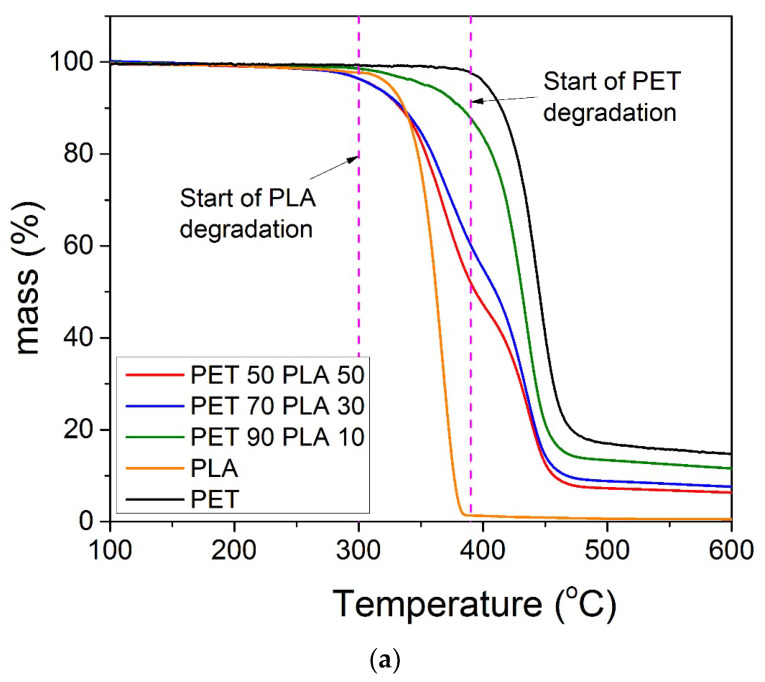

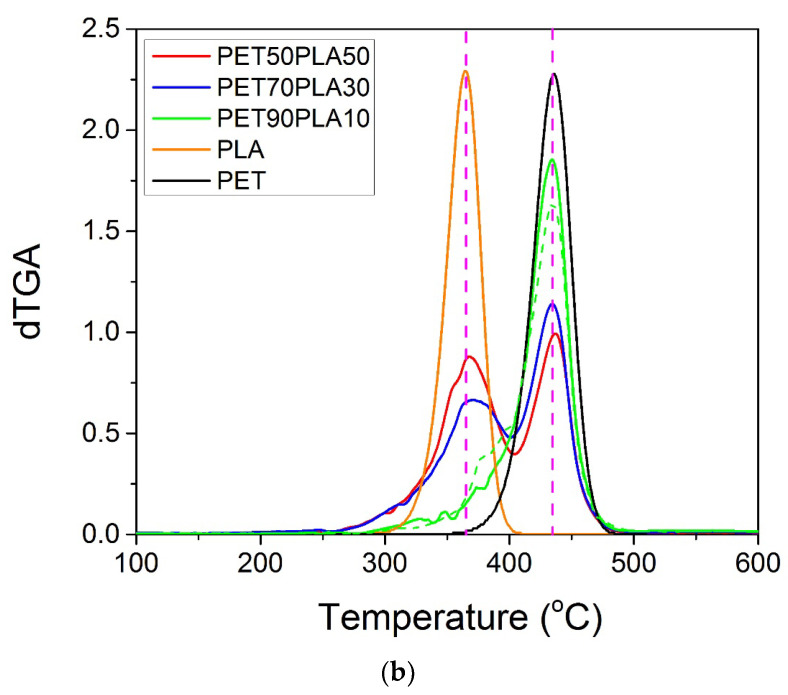
TG scans of the neat rPET and rPLA and their blends at a heating rate of 10 °C/min (**a**) and corresponding derivative TG of all samples (**b**).

TG scans for the neat rPLA, rPET, and their blends together with the derivative of the corresponding TG scans appear in Figure 7a,b. It can be seen that PLA degrades at a much lower temperature compared to PET. Specifically, rPLA starts degrading at around 300 °C, whereas the onset of rPET degradation is at almost 380 °C, in accordance with the literature values [23]. Degradation of the PET/PLA 50/50 and 70/30 blends starts at slightly lower temperatures compared to the neat rPLA, at around 276–277 °C, with the blend richer in PET showing an onset temperature in between those of rPLA and rPET (i.e., 316 °C). It has been proposed that the degradation of PLA is promoted by ester interchange reactions, whereas random chain-scission via a cis-elimination is the dominant mechanism [36,37,38]. Therefore, the first step of the blends’ degradation may be attributed to ester interchange reactions due to the presence of PET. In addition, the thermal degradation curves of all neat polymers exhibit one step, whereas those of all blends clearly show two steps in the temperature region between that of rPLA and rPET. The first degradation step is attributed to the thermal degradation of rPLA, whereas the second is attributed to that of rPET. As was expected, higher amounts of rPET in the blend provide better thermal stability to the material. Therefore, it was verified that contamination of rPET with rPLA results in a product with poor thermal stability relative to rPET. Very interesting points also come from observation of the dTGA curves and the two dominant peaks appearing. It was clear (Table 3) that the second peak, attributed to the degradation of rPET, was almost constant in all materials tested and near to 435 ± 1 °C. The temperature at the maximum rate of degradation of rPET is also 435 °C. This means that the second peak in the derivative curves is clearly due to rPET degradation. However, concerning the first peak, the temperature shows a gradual increase from 368 to 380 °C with the increasing percentage of rPET in the blend. The maximum rate degradation temperature of the neat rPLA was measured at 367 °C. Therefore, it seems that at relative amounts of rPLA greater than those of rPET in the blend, rPLA keeps its identity and behaves like a neat rPLA. However, when rPET is in larger quantities, the presence of this polymer in the blend acts as a kind of barrier on the one hand to the heat transfer inside the material and on the other to the evaporation and removal of volatile compounds from the decomposition of rPLA. All of these factors result in shifting the degradation of rPLA to higher temperatures. A similar trend was observed in all other materials studied here.

The residual masses of rPLA and rPET at 600 °C were 0.5 and 15.1%, respectively. The aliphatic nature of the former polymer results in much lower residual mass compared to the aromatic structure of the latter. The blends show residual masses in between those limit values, i.e., 6.3, 7.8, and 11.6% for relative amounts of 50/50, 70/30, and 90/10, respectively. If one considers the additive rule, then the theoretical values would be 7.6, 10.4, and 13.3%, respectively. All of the experimental values were lower compared to the theoretical ones.

**Figure 8:**
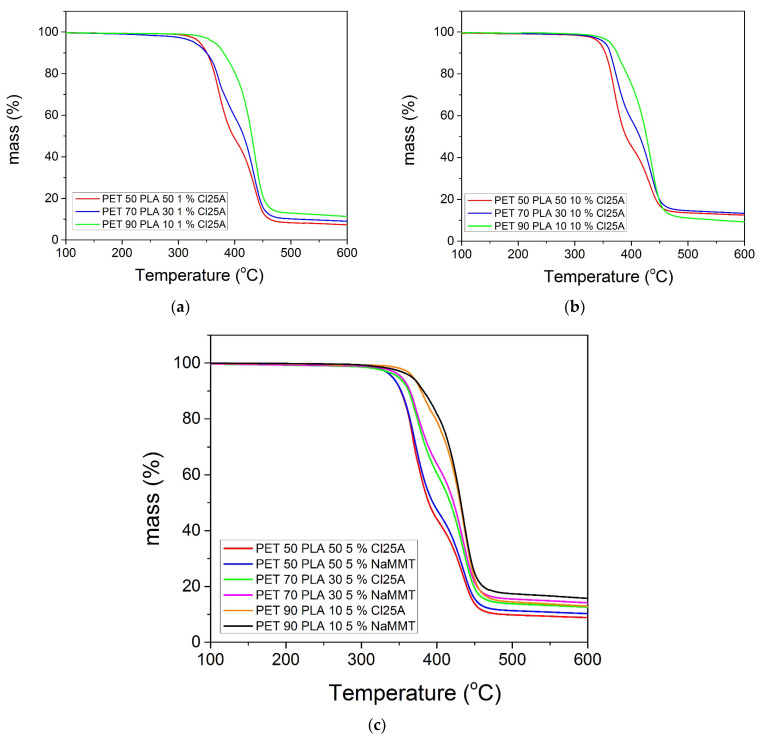
TG scans of the PET/PLA blends at a heating rate of 10 °C/min: (**a**) with 1 wt% Cloisite 25A; (**b**) 10 wt% Cloisite 25A; and (**c**) 5 wt% Cloisite 25A and 5 wt% Na+MMT clay.

**Figure 9:**
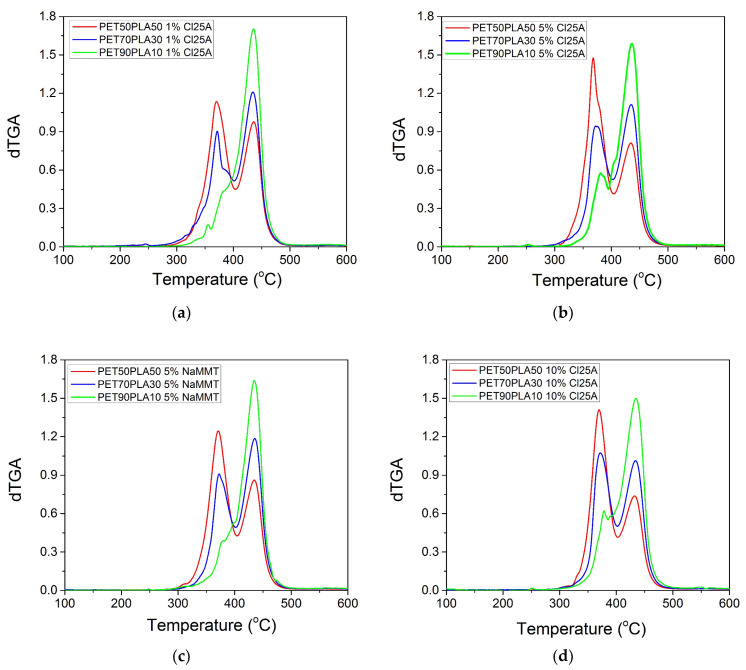
Derivative TG scans of the PET/PLA blends with: (**a**) 1 wt% Cloisite 25A; (**b**) 5 wt% Cloisite 25A; (**c**) 5 wt% Na+MMT; and (**d**) 10 wt% Cloisite 25A.

Furthermore, the results of the mass loss with temperature for the nanocomposite blends with 1 and 10% Cloisite 25A appear in Figure 8a,b, respectively. The nanocomposites with 5% nanoclay, either Cloisite 25A or Na^+^MMT, are included in Figure 8c. The corresponding derivative TG curves are illustrated in Figure 9a–d, for the same materials. Concerning the effect of the relative amount of rPET to rPLA, exactly the same observations with those previously reported for the neat blend were clear. Specifically, all nanocomposite blends show degradation in two steps, with temperatures shifted to higher values with the increasing amount of rPET in the blend. In the derivative, dTGA, for the plots in all cases, the area of the first peak related to rPLA degradation decreased with the decrease in the amount of rPLA accompanied by an increase in the area of the peak related to rPET with the increasing amount of rPET.

The effect of the amount of nanoclay on the thermal degradation of each blend investigated is illustrated in Table 3. It can be seen that the onset of mass loss compared to the neat material shifted to higher temperatures for each blend as 1% or 5% nanoclay was added. However, the thermal degradation behavior of the material with 10% nanoclay was in most cases similar or slightly different compared to that with 5%. This is an indication that, as has been observed with the WAXD measurements, agglomerates may be formed when high amounts of nanoparticles are added.

The residual amount of the three blends at 600 °C was 7.3, 9.0, and 11.3% for the composites with 1% nanoclay. Compared with the amount measured for the neat blend, i.e., 6.3, 7.8, and 10.4%, increases of 1, 1.2, and 0.9% were noticed, respectively, which are near to the nominal amounts of the nanoclay added. This again is an indication of good dispersion of the montmorillonite in the polymer matrix. The residual masses measured with the 5 wt% Cloisite 25A were 8.8, 12.6, and 12.9 for the bends with 50/50, 70/30, and 90/10 rPET/rPLA, respectively. These values correspond to an increase of2.5, 4.8, and 2.5%, respectively. The corresponding values estimated when 5% of Na+ MMT was added were 4.0, 6.2, and 5.3 wt%, respectively. The larger value of the latter case is due to the poor dispersion of this MMT in the polymer matrix as well as due to the fact that part of the organo-modification of the org-clay near 25% also degraded at 600 °C, whereas the sodium clay did not degrade significantly at this temperature. The shifting of the degradation curve to higher temperatures with the nanoclay added is attributed to the fact that the nanoclay acts as a barrier, retarding the diffusion of the volatile degradation products out of the polymer.

### 3.3. Thermal Properties of the Blends and the Nanocomposites

**Figure 10:**
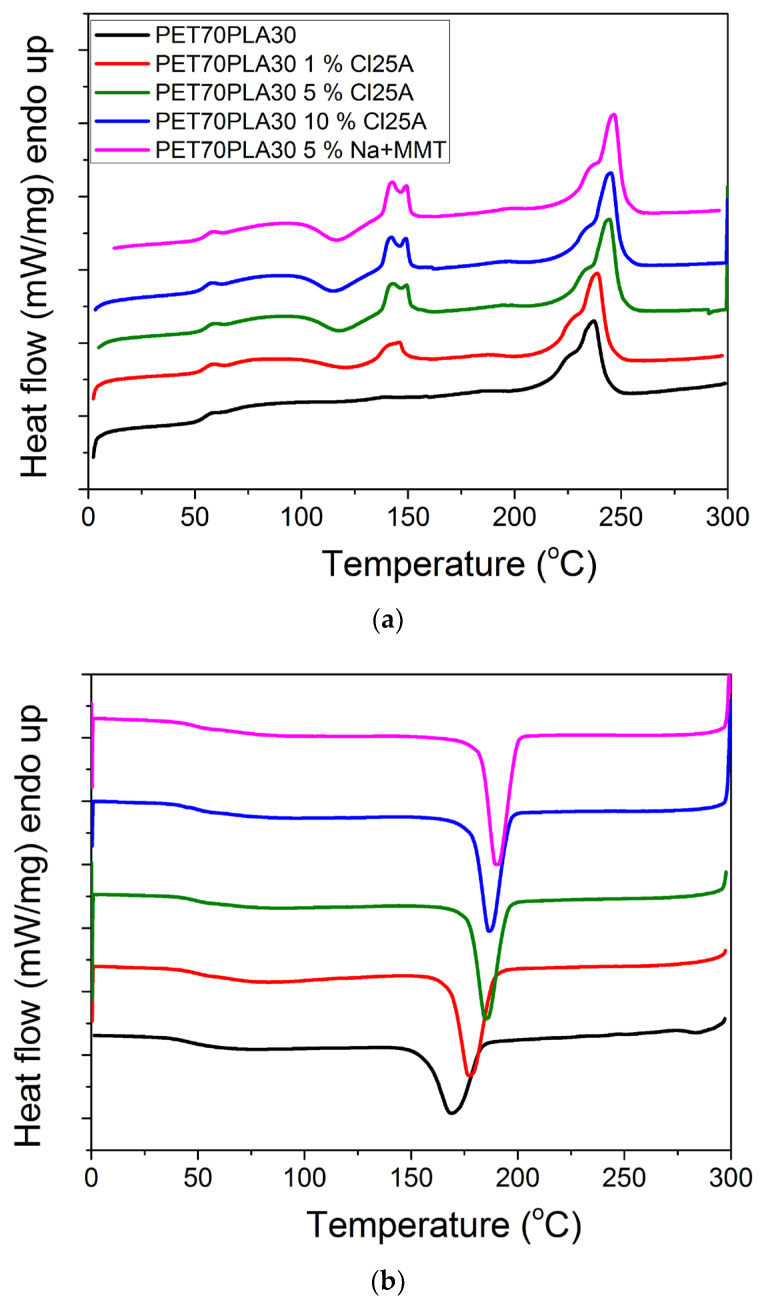
DSC scans showing the effect of the amount of clay on the heat flow measured during heating (**a**) or cooling (**b**) of the nanocomposite blends with the composition of 70-30.

**Figure 11:**
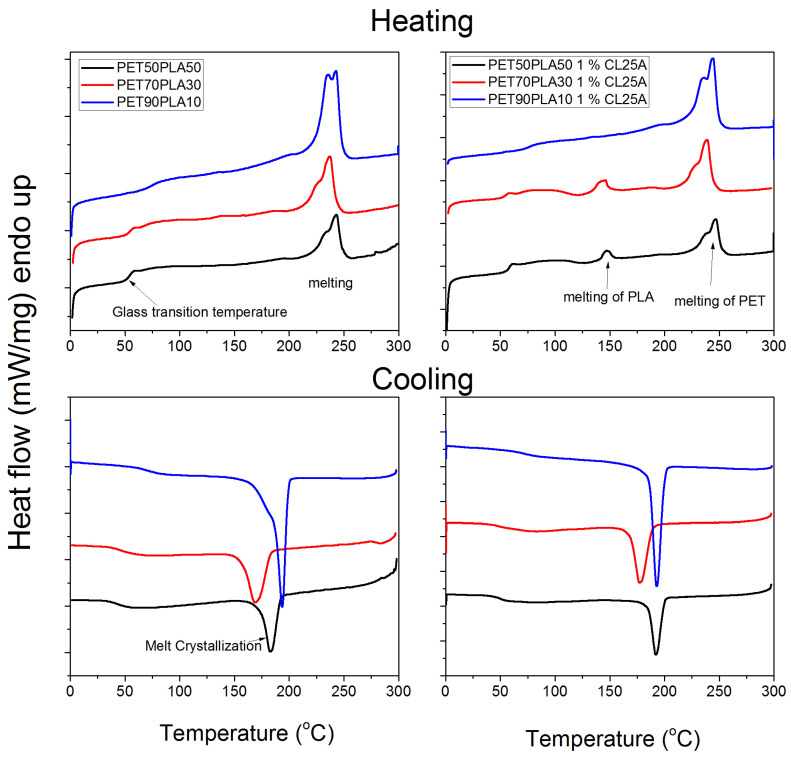
DSC curves measured during heating or cooling showing the effect of the relative amount of PET /PLA on the thermal properties of the blends.

Finally, DSC measurements were carried out to estimate the thermal properties of the blends investigated. Indicative plots on the effect of the nanoclay on the heat flow recorded during heating or cooling appear in Figure 10a,b. Moreover, DSC scans showing the effect of the relative amount of the PET/PLA are illustrated in Figure 11. All results are included in Table 4.

During heating, the glass transition temperature of the material was estimated together with the melting temperature of the PET domain mainly and in some blends of the PLA. During cooling, the crystallization temperature and enthalpy were measured. Since the glass transition temperature of PET is higher than that of PLA, as the amount of PET increases in the blends, a slightly higher Tg of the blend was measured. The existence of the nanoclay results in slightly higher Tg values. Moreover, the melting temperature of the PET domain is not affected by the presence of the nanoclay much. The enthalpy of melting of the 50/50 blends is lower than that of the corresponding 70/30 and 90/10. This means that the degree of crystallinity of these blends is lower compared to the other ratios in accordance with the XRD measurements. The amount of nanoclay shifts the crystallization temperature to higher values, denoting their effect as a heterogeneous nucleating agent in the system.

## 4. Conclusions

Several blends of PET and PLA taken from recycled bottles and glasses were prepared via melt mixing, with compositions ranging from 50-50 to 90-10. Their morphological and thermal properties were studied via several methods. Based on SEM measurements, it was found that in the 50-50 blends, the dispersion of rPLA into rPET was homogeneous, whereas in higher amounts of rPET, large parts of rPLA seem to be included into the rPET matrix. A slight decrease in roughness with the increasing amount of rPET in the polymer blend was observed in the AFM. Concerning the relative crystallinity, the blends keep the main characteristics of the neat polymers, as revealed by the XRD scans. According to the TGA thermograms, thermal degradation of the blends occurs in two steps in accordance with the degradation of the individual polymers. An increasing amount of PLA in the blends shifts the onset of degradation to lower temperatures. From the DSC measurements, an increase in Tg with the increasing amount of PET and nanoclay in the blends was observed. The melting temperature of the PET domain was not significantly affected, whereas the enthalpy of melting revealed that the increasing amount of PET resulted in a higher degree of crystallinity. The existence of the nanoclay acts as a nucleating agent.

Additionally, the nanocomposites were prepared based on these blends, with 1, 5, and 10% organomodified commercial clay and 5% natural Na MMT. The clay was found to have good dispersion in the polymer matrix while its amount increased the roughness of the materials. The XRD measurements revealed mainly intercalated structures up to 5% with partially exfoliated structures at 1%. However, at higher amounts of nanoclay loading, i.e., 10%, agglomerates are formed. An increasing amount of nanofiller results in increased roughness of the materials. The thermal degradation was increased by the presence of the nanoclay following the increasing amount of filler.

Biopolymers will continue to be found in plastic waste in the near future, so we must be ready to start collecting them individually as soon as possible. Mixed waste must be recycled simultaneously until then, and an approach has to be discovered for this. Since many papers have already examined the differences in the properties of PET and PLA separately during recycling, we focused on the qualities of the mixes in our research. In this case, we also looked for a way to recycle mixed PET and PLA bottles more effectively, as well as a way to increase their durability and other characteristics by adding various amounts of clay. Therefore, new materials with improved properties may be prepared using mixed polyester waste.

## Figures and Tables

**Table 1:** Composition of rPET and rPLA in the prepared blends together with the amount of nanoclay used.

Sample	rPET (wt%)	rPLA (wt%)	Cloisite 25A (wt%)	Na + Montmorillonite (wt%)
PET50PLA50	50	50	-	-
PET50PLA50 1% Cl25A	50	50	1	-
PET50PLA50 5% Cl25A	50	50	5	-
PET50PLA50 10% Cl25A	50	50	10	-
PET50PLA50 5% NaMMT	50	50	-	5
PET70PLA30	70	30	-	-
PET70PLA30 1% Cl25A	70	30	1	-
PET70PLA30 5% Cl25A	70	30	5	-
PET70PLA30 10% Cl25A	70	30	10	-
PET70PLA30 5% NaMMT	70	30	-	5
PET90PLA10	90	10	-	-
PET90PLA10 1% Cl25A	90	10	1	-
PET90PLA10 5% Cl25A	90	10	5	-
PET90PLA10 10% Cl25A	90	10	10	-
PET90PLA10 5% NaMMT	90	10	-	5

**Table 2:** Roughness parameters (arithmetical mean deviation from the mean, Sa, and root mean square (RMS) mean deviation for the mean, Sq) obtained from AFM measurements of all PET/PLA blends and nanocomposites with the nanoclayes studied.

Sample	Roughness, Sa (nm)	RMS, Sq (nm)
PET50PLA50	12,758	17,387
PET50PLA50 1% Cl25A	12,370	19,553
PET50PLA50 5% Cl25A	20,388	27,810
PET50PLA50 10% Cl25A	82,256	100,144
PET50PLA50 5% NaMMT	16,163	22,406
PET70PLA30	11,581	14,138
PET70PLA30 1% Cl25A	21,525	27,120
PET70PLA30 5% Cl25A	71,848	97,708
PET70PLA30 10% Cl25A	84,600	116,233
PET70PLA30 5% NaMMT	67,444	92,453
PET90PLA10	11,022	15,018
PET90PLA10 1% Cl25A	14,023	18,065
PET90PLA10 5% Cl25A	25,159	36,664
PET90PLA10 10% Cl25A	78,898	108,502
PET90PLA10 5% NaMMT	23,505	32,392

**Table 3:** TG measurements of the characteristic temperatures for the thermal degradation of rPLA, rPET, and the nanocomposite blends. Onset of thermal degradation: T_2%_, temperature of the first: T_p1_, and the second: T_p2_ peak and temperature at the end of degradation: T_end_ together with residual mass measured at 600 °C.

Sample	T_2%_ (°C)	T_p1_ (°C)	T_p2_ (°C)	T_end_ (°C)	Residual at 600 °C
rPLA	315	367		391	0.5
rPET	407		435	484	15.1
PET50PLA50	276	368	435	490	6.3
PET50PLA50 1% Cl25A	308	369	435	491	7.3
PET50PLA50 5% Cl25A	324	369	435	491	8.8
PET50PLA50 10% Cl25A	318	369	434	492	12.5
PET50PLA50 5% NaMMT	322	370	434	492	10.3
PET70PLA30	277	371	435	495	7.8
PET70PLA30 1% Cl25A	310	372	435	496	9.0
PET70PLA30 5% Cl25A	317	373	435	496	11.6
PET70PLA30 10% Cl25A	324	372	434	497	13.3
PET70PLA30 5% NaMMT	330	372	436	496	14.0
PET90PLA10	316	380	435	492	10.4
PET90PLA10 1% Cl25A	338	382	435	495	11.3
PET90PLA10 5% Cl25A	341	382	436	495	12.9
PET90PLA10 10% Cl25A	339	380	434	495	9.2
PET90PLA10 5% NaMMT	338	380	435	495	15.7

**Table 4:** DSC measurements of the thermal properties of the nanocomposite blends. Glass transition temperature: T_g_, melting temperature of the PET domain: T_m_, corresponding enthalpy of melting: ΔH_m_, crystallization temperature: T_c_, and the enthalpy of crystallization from the melt, ΔH_c_.

Sample	T_g_ (°C)	T_m_ (°C)	ΔH_m_ (^J/g^)	T_c_ (°C)	ΔH_c_ (J/g)
PET50PLA50	55	249	15.1	206	17.0
PET50PLA50 1% Cl25A	59	249	17.5	197	15.5
PET50PLA50 5% Cl25A	59	249	15.2	205	15.8
PET50PLA50 10% Cl25A	59	249	12.3	203	19.2
PET50PLA50 5% NaMMT	60	249	19.4	203	19.9
PET70PLA30	54	248	31.8	202	31.2
PET70PLA30 1% Cl25A	54	243	28.4	196	28.1
PET70PLA30 5% Cl25A	57	247	22.1	200	21.3
PET70PLA30 10% Cl25A	59	248	23.8	201	24.4
PET70PLA30 5% NaMMT	59	248	27.3	204	28.0
PET90PLA10	63	249	34.1	203	34.2
PET90PLA10 1% Cl25A	67	250	33.6	201	33.4
PET90PLA10 5% Cl25A	67	249	36.1	206	33.8
PET90PLA10 10% Cl25A	68	250	32.4	208	28.1
PET90PLA10 5% NaMMT	65	247	33.0	208	33.6

## Data Availability

Data are available upon request.
